# Estimating the Total Number of Susceptibility Variants Underlying Complex Diseases from Genome-Wide Association Studies

**DOI:** 10.1371/journal.pone.0013898

**Published:** 2010-11-17

**Authors:** Hon-Cheong So, Benjamin H. K. Yip, Pak Chung Sham

**Affiliations:** 1 Department of Psychiatry, University of Hong Kong, Hong Kong SAR, China; 2 Genome Research Centre, University of Hong Kong, Hong Kong SAR, China; 3 The State Key Laboratory of Brain and Cognitive Sciences, University of Hong Kong, Hong Kong SAR, China; Peninsula Medical School, United Kingdom

## Abstract

Recently genome-wide association studies (GWAS) have identified numerous susceptibility variants for complex diseases. In this study we proposed several approaches to estimate the total number of variants underlying these diseases. We assume that the variance explained by genetic markers (Vg) follow an exponential distribution, which is justified by previous studies on theories of adaptation. Our aim is to fit the observed distribution of Vg from GWAS to its theoretical distribution. The number of variants is obtained by the heritability divided by the estimated mean of the exponential distribution. In practice, due to limited sample sizes, there is insufficient power to detect variants with small effects. Therefore the power was taken into account in fitting. Besides considering the most significant variants, we also tried to relax the significance threshold, allowing more markers to be fitted. The effects of false positive variants were removed by considering the local false discovery rates. In addition, we developed an alternative approach by directly fitting the *z*-statistics from GWAS to its theoretical distribution. In all cases, the “winner's curse” effect was corrected analytically. Confidence intervals were also derived. Simulations were performed to compare and verify the performance of different estimators (which incorporates various means of winner's curse correction) and the coverage of the proposed analytic confidence intervals. Our methodology only requires summary statistics and is able to handle both binary and continuous traits. Finally we applied the methods to a few real disease examples (lipid traits, type 2 diabetes and Crohn's disease) and estimated that hundreds to nearly a thousand variants underlie these traits.

## Introduction

The number of genome-wide association studies (GWAS) has grown rapidly in the past few years [1]. GWAS have identified a number of robust associations for complex diseases like breast cancer, prostate cancer, type 1 and 2 diabetes etc. As more variants are discovered, it is natural to ask: what is the *total* number of susceptibility variants that underlie these complex diseases? Or equivalently, how many more variants need to be found in order to explain the entire heritability?

It turns out that the results from GWAS could provide important clues to the above questions. We developed a methodology to tackle the problem by fitting distributions to the GWAS results. We assumed that the effect sizes of all susceptibility variants in the genome, as measured by the variance explained (Vg), follow an exponential distribution. For binary traits, the variance explained is computed based on the liability threshold model. The model proposes a latent continuous liability, which is assumed to follow a normal distribution with mean 0 and variance 1. The variance in liability explained can be directly interpreted as the locus-specific heritability. The method is described in details in another paper [2].

Our aim is to fit the observed distribution of Vg from GWAS to its theoretical distribution. In practice, the sample size of a study is limited and there is inadequate power to detect variants with small effect sizes. As a result, variants with larger effects are over-represented and the Vg of the discovered variants would not follow a standard exponential curve. To correct for this bias, we also considered the statistical power corresponding to every given Vg. In addition, we developed an alternative estimator of the number of variants by directly fitting a suitable distribution to the observed *z*-statistics.

The assumption of an exponential distribution of effect sizes is theoretically justified. Orr [3] studied Fisher's model of adaptation and considered random adaptive walks to the optimum. He showed that the sizes of the factors fixed during adaptation assume an exponential distribution. The exponential model was also supported by a paper by Bost *et al.* [4], in which fluxes through metabolic pathways were studied.

The problem of the total number of variants in the genome is rarely addressed. Recently a study by Pawitan *et al.* [5] asked a similar question. However, their methods and focus is largely different from our current study. In their study, they have constructed several hypothetical scenarios of allele frequencies and odds ratios (ORs), and estimated the number of variants required to make up a total 40% of heritability. Assuming a pattern of allele frequencies and ORs “similar” to what has been observed for confirmed variants, they also estimated the number of susceptibility variants for type 2 diabetes. However the details of calculations were not shown. They also employed variance in liability explained as an effect size measure, which is similar to our study (but we assume a normal distribution of the underlying liability rather than a logistic distribution). In contrast to Pawitan *et al.*, we did not construct specific combinations of allele frequencies and ORs, instead we fit appropriate distributions to actual GWAS data to estimate the number of variants. The array of methodologies presented in this paper has not been previously described.

At the time of submission, we also noticed an interesting paper by Park et al. [6] which have addressed a similar problem. However, the problem addressed in this study is *not* exactly the same as in Park et al. We are considering the *entire* range of effect sizes while in Park et al the range of effect sizes is limited to those observed for known susceptibility variants. In other words, Park et al. aimed at estimating the total number of variants *within the range of effect sizes that have been observed in GWAS conducted to date*. On the other hand, we aimed at providing a framework to *evaluate the total number of variants underlying a disease, regardless of the effect sizes of variants*. The number of variants estimated from Park et al. will therefore be smaller than the number from our approach. For example, for Crohn's disease, Park et al. estimated the number of loci to be ∼150 within the range of effect sizes of known loci while our estimate is around a thousand (as detailed in later sections). Park et al [6] also addressed issues of power calculations for future GWAS and estimate predictive power of common variants using AUC (area under the curve of the receiver operating characteristic curve). Our study is more focused on exploring various approaches to estimation of the number of variants and tackling the complications involved, such as different ways to correct for winner's curse, relaxing the significance threshold with correction for false positives and deriving the confidence intervals analytically. We have also perfozrmed extensive simulations to compare and verify the performance of totally fourteen different proposed estimators.

## Methods

### Exponential distribution of Vg for all susceptibility variants in the genome

We assume that the Vg of all susceptibility variants in the genome follow an exponential distribution. The probability density function (pdf) of an exponential distribution can be expressed as
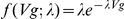
where the rate *λ* is the only parameter that characterize the distribution. The mean is simply given by 1/*λ*. Therefore the number of susceptibility variants can be derived given *λ*:
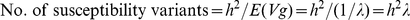
where 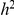 is the total heritability.

### Probability density function for Vg in GWAS and fitting by maximum likelihood

**Figure 1:**
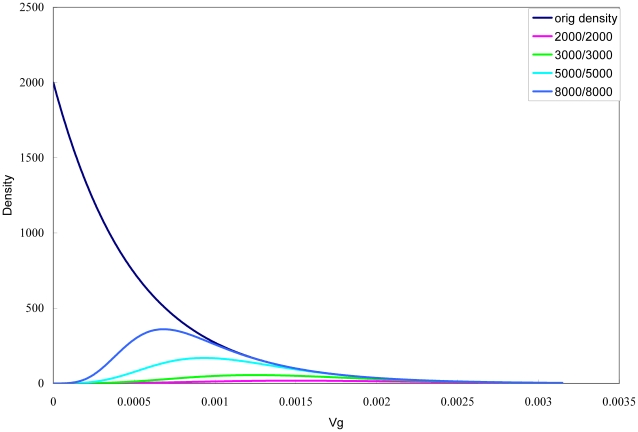
Probability density of the Vg of detected variants in a GWAS with adjustment for power. We assume an exponential distribution of Vg for susceptibility variants under unlimited sample size. In practice, sample size and power is limited and small-effect variants will be under-represented. Therefore the probability density should be adjusted for power. “Orig density” denotes the original exponential distribution, and the numbers like 2000/2000 denotes the number of cases and controls. The significance threshold was set at 5×10^−7^ and prevalence set at 0.001. Lambda equals 2000. The risk allele frequency was assumed at 0.5.

In practice one cannot observe all the susceptibility variants. Suppose a GWAS was performed and some variants were found to be associated with *p*-values below a certain threshold *α*. The probability density of the Vg of these observed significant variants is directly proportional to the standard exponential density multiplied by the power:
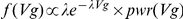
where *pwr* is a function that returns the corresponding power for a given Vg, assuming allele frequency, prevalence, type I error (*p*-value cutoff) and sample size are all fixed. Examples of the power-adjusted pdf for different sample sizes are shown in Figure 1. The power was evaluated based on a simple approach described in detail in So and Sham [7]. Briefly, we computed z = ln(OR)/SE(ln OR) under H_1_ and estimated the corresponding power. Here we take advantage of the fact that the power corresponding to a given Vg is grossly similar regardless of the risk allele frequency (see Table S1 for some examples). The allele frequency was therefore not taken into account for power calculation but was set at a fixed value (0.5). The problem will however be significantly complicated if we do consider the modest difference in power for different allele frequencies, as the above simple formulation of Vg distribution can no longer be applied. For a continuous outcome, the exact power can be derived by the variance explained alone (see later sections).

To scale the above density so that it integrates to 1, we divide the above density by the normalizing factor 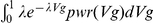. Hence the probability density function (pdf) of Vg, corrected for inadequate coverage of small-effect variants due to limited sample size, is given by
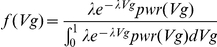
Note that the denominator is only a function of *λ* and is not a function of Vg since Vg is integrated over its possible range.

The above pdf can be used for maximum likelihood estimation of *λ* from a list of “significant” Vg obtained from genome-wide association studies. 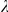 can be obtained by maximizing the following log-likelihood function:
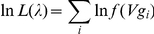
where *i* refers to the *i*th observation. The details of deriving the maximum likelihood estimate (MLE) can be found in the appendix.

### Distribution fitting by methods of moments

The theoretical population mean of Vg under the power*density curve is given by
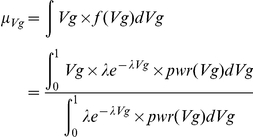
We can then find the *λ* that gives the closest theoretical mean to the observed sample mean of Vg from GWAS. In other words, we solve the following equation,
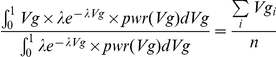
It turns out that the method of moments and maximum likelihood give identical estimates for *λ* (the proof is given in Text S1). Therefore we can use either method for distribution fitting. The above formulation given by methods of moments is computationally simple.

### Relaxing the *p*-value threshold

Typically in GWAS we only consider the variants that withstand a Bonferroni correction (typical threshold = 0.05/total no. of variants) to be significant. However, for fitting the density curve and estimation of *λ*, a small number of false positives may be allowed to reduce variance. Here we allow a more liberal threshold so that more variants can be used in distribution fitting and estimation of *λ*. At the same time, we make use of the local false discovery rate (lfdr) procedure [8] to remove the effect of false positive variants, so that the estimated mean reflect only the effects of truly associated variants.

The local fdr is the posterior probability of H_0_ given the test statistic.
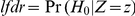
The posterior probability of H_1_ given the test statistic, or Pr(H_1_|Z = z), is given by (1-lfdr). Local fdr is calculated for the *z*-value of every SNP (e.g. from Wald test in logistic regression) in a GWAS. The Vg for each SNP is weighted by 1-lfdr corresponding to that particular SNP. The weighted mean of Vg can be expressed by
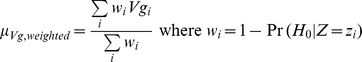



Although theoretically we may include all SNPs in distribution fitting, it should be noted that the local fdr around *z* = 0 is often close to or equal to 1, due to limited sample size. The actual number of truly associated variants is usually larger than that predicted by local fdr when *z* is around 0. Inclusion of all variants will lead to an underestimation of mean Vg, as the weights for SNPs with *z*-values around 0 will be inappropriately low. Therefore we set an fdr threshold and only considered *z*-values corresponding to a fdr lower than a particular value. Here we assume that when we restrict our consideration to *z*-values below the fdr threshold, we can accurately assess the non-null density of *z* (denoted f_1_(*z*) by Efron), and hence “recover” the Vg correponding to these non-null *z*-values. The statistical power (required for the construction of the power x density curve) is calculated according to the *z*-value cutoff. Local fdr was calculated by the locfdr program by Efron [9].

### Fitting the most significant *z*-values to the alternative density

Another approach is to consider 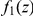, the density of the non-null *z*-statistics. Since we assume the Vg of associated variants follow an exponential distribution with parameter *λ*, we can derive 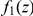 which is also characterized by the parameter *λ*. We may then fit the most significant *z*-values to 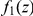, and estimate the underlying *λ*. This is similar to our earlier proposal of fitting observed Vg of significant markers to the power x density curve, but this time the *z*-values instead of Vg are used for distribution fitting.

#### Evaluating the alternative density of z

Note that the observed z-values do not equal the true z-values. Denoting the true z-value by δ, then
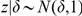
Alternatively, we may consider an observed *z*-value as a sum of the true *z*-value and a random “noise” component which is standard normal:
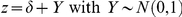
We first consider the distribution of *δ*. For simplicity, we first restrict all *δ* to be positive and denote them by *δ^+^*. Let g be a function that converts Vg to *δ*, ie
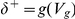
(since *δ* are all positive, a single Vg can only give a single value of *δ*, in other words, there is a one-to-one correspondence of Vg and *δ*)

The density of δ is given by
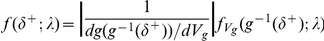
where 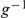 is a function converting *δ^+^* to Vg and 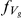 is the density of Vg. 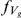 is an exponential distribution characterized by the parameter *λ*.

Now we consider all values of δ, positive and negative. The distribution of *δ* is symmetrical i.e. *f*(*δ*) = *f*(−*δ*), so the above densities are twice of the overall density which includes all values of *δ*. The overall density of *δ* is given by
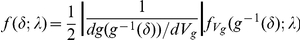



Now we turn to the evaluation of 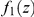.Since we have
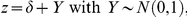
we can obtain 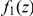 by convolution of the density functions of *δ* and *Y*:
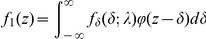
Note that *f*_1_(*z*) is determined by parameter *λ* only as we integrate over *δ*.

#### Fitting observed data to the alternative density

Since the sample size is limited, we can only detect variants with effect sizes larger than a certain extent. Assuming a Bonferroni correction is used and family-wise error rate is set at *α*, the *z*-value threshold *z_crit_* is given by 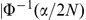 where *N* is the total number of markers in the study. The observed significant *z*-values hence follows a truncated 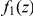
with truncation at *z_crit_* and - *z_crit_*. The pdf of the truncated 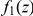 is given by
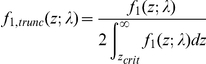
assuming symmetrical distribution of effect sizes, i.e. 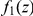 = 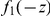, such that the truncated area on the left and the right are equal.

The log likelihood for a set of significant *z*-values is
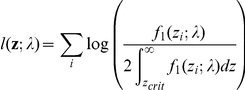
The MLE of *λ* can be obtained by numerical maximization of the above likelihood function.

Again one may wish to relax the significance threshold to allow more variants for distribution fitting. To remove the effects of the false positive variants, we may employ a weighted likelihood approach with weights equal to Pr(H_1_) for each included marker. Pr(H_1_) may be estimated by 1 minus the local fdr for each marker. The weighted likelihood is
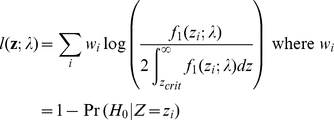
For reasons already described above, we set an fdr threshold and only include SNPs below that threshold.

By fitting the distribution of the truncated 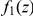, there is no need to further correct for winner's curse because we have already conditioned on 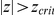. (Since we assume symmetric distribution of effect sizes, 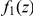 = 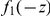 and in practice one may work with 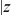 instead.)

### Correcting for winner's curse

Winner's curse refers to the possible overestimation of effect size of *significant* genetic variants in association studies. It may be interpreted as a form of selection bias. The selection of SNPs meeting significance threshold alters the probability distribution of odds ratios (or *z*-values) for the chosen SNPs, resulting in bias in effect size estimates. The selection bias is most prominent when the study has weak power and when the significance threshold is extreme. This is particularly pertinent to genome-wide association studies, in which the association signals are usually weak and very stringent significance thresholds are often imposed to guard against multiple testing. As overestimation of effect size will lead to overestimation of mean Vg, or underestimation of *λ*, we investigated various statistical methods based on conditional likelihood to correct for the winner's curse.

The details of the winner's curse correction approaches applied here were described by Ghosh *et al.* [10] and Zhong & Prentice [11]. We consider the test statistic for a single marker in the following form:
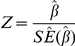
The above statistic is assumed to be asymptotically normal. For example, when one performs logistic regression to test for SNP associations, the Wald test statistic will take the above form. 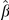 then denotes the estimated regression coefficient.

In our case, we are only interested in SNPs with *z*-values exceeding a certain threshold *c*, where *c* is chosen such that the family-wise error rate (FWER) or false discovery rate are controlled below a certain level. The selection of SNPs passing the significance cutoff distorts the original normal distribution. The conditional likelihood after selection can be expressed by [10], [12]

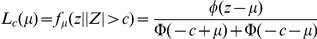



Instead of working with the *z*-values, Zhong and Prentice [11] worked with the regression coefficients *β* and used an equivalent form of the above conditional likelihood in their paper. Here for clarity, we focused on the formulation using the *z*-statistics. A number of corrected estimates of the effect size have been proposed based on the above result. Below we briefly describe five estimators considered in our simulations.

The first one is the MLE estimator
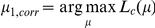
which is in fact the same as matching the expectation of the sampling distribution of *μ* to the observed mean.

The second one is the mean of the normalized conditional likelihood,
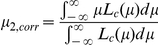
which aims to reduce the mean squared error from a Bayesian point of view. 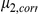 may be treated as a posterior mean with a flat prior on *μ*.

The third estimator is the mean of the first two estimators
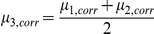
which aims to combine the strength of the first and second estimators.

The fourth estimator produces a sampling distribution of *z* with median at the *observed z*-value. The estimator can be expressed as
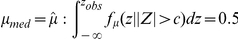
where *z*_obs_ is the observed *z*-statistic for the SNP under study. Note that Zhong and Prentice [11] dealt with the distribution of regression coefficient *β*, but since *z* and *β* are directly related through the fixed SE (*z* = *β*/SE), it does not make any difference if one works with the sampling distribution of *z*.

The fifth estimator is a weighted average of the uncorrected regression coefficient estimate (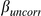) and the median corrected coefficient estimate (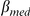) (note 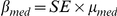). The weight depends on the estimated variance of the uncorrected coefficient (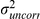) and the difference between the corrected and uncorrected coefficients.
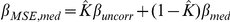
where 
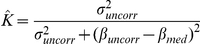
.

All the above correction methods work for binary as well as continuous outcomes. The first four methods just require *z*-values as input. For the last method, an estimate of the standard error of coefficient is required. As a crude approach, we again assumed a fixed risk allele frequency of 0.5 so that log(OR) and the standard error (SE) can be estimated from *z*-values. It should be noted that the SE is in fact a function of the risk allele frequency. For the same level of relative risk and the same z-statistic, a larger SE will result if risk allele frequency is lower. Ignoring the risk allele frequency will result in less accurate estimates of SE. Hence it is recommended that one considers the actual risk allele frequencies if they are available. In view of this limitation, the results that are based on the last correction method (i.e the “MSEmedian” estimators) should be regarded as rough estimates only.

### Simulation strategy

We simulated *z*-statistics and investigated the performance of the various estimators in recovering the underlying parameter that generates the statistics. The *z*-statistics were composed of two groups: the first group corresponded to H_0_ with distribution *N*(0,1) ; the second group corresponded to H_1_ (i.e. truly associated variants) with distribution *N*(*δ*,1), where *δ* was determined by the parameter of the distribution of Vg as described below.

We simulated the Vg of associated variants according to the exponential distribution,
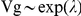
Given the allele frequency, prevalence and sample size, one may find the relative risk that produces the given Vg and hence a corresponding *z*-value (denoted δ) can be obtained. Since the same Vg gives rise to similar power and *z*-values, we simply fix the allele frequency at 0.5.

In each replication we simulated 100,000 *z*-values, 99.5% of which are null. The non-null *z*-values were derived from their corresponding exponentially distributed Vg. We assumed a prevalence of 0.001 and a local fdr cut-off of 0.3 throughout. We investigated the performance of a number estimators under three different sample sizes (number of cases = 3500, 5000 and 7000 with equal number of controls) and four different *λ* (1000, 2000, 3000, 4000). The root mean squared error from 300 simulations was computed. The root mean squared error is given by
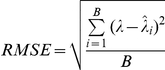
where *B* is the number of simulations, 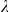 is the true parameter value and 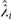 is the estimated parameter value in the *i* th simulation.

In total fourteen estimators of *λ* were evaluated. Table 1 provides a summary of the 14 estimators under study. The estimators fell into two main groups. The first group of estimators was obtained from fitting only the variants passing a Bonferroni correction. The family-wise error rate was set at 0.05. The second group of estimators were obtained from fitting all variants below an fdr threshold (set at 0.3), which allowed inclusion of an increased number of markers. In each group, five estimators were obtained by first applying different winner's curse correction methods to the observed effect sizes and then fitting a density x power curve to the corrected levels of Vg. For comparison, we also included in each group an estimator derived from fitting the density x power curve but *without* any winner's curse corrections. Lastly, we fit the truncated convolution density 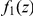 to the selected *z*-values in each group.

**Table 1:** An overview of the proposed estimators of *λ*.

Name of estimator	SNP inclusion criteria	Distribution fitting method	Winner's curse correction	Corresponding numbering in figures
Bonf	Bonf	pwr*exp_density	None	1
Bonf.corr	Bonf	pwr*exp_density	Avg of Ghosh 1&2	2
Bonf.corr1	Bonf	pwr*exp_density	Ghosh 1	3
Bonf.corr2	Bonf	pwr*exp_density	Ghosh 2	4
Bonf.corr.med	Bonf	pwr*exp_density	Median (Zhong)	5
Bonf.corr.MSEmedian	Bonf	pwr*exp_density	MSE median (Zhong)	6
Bonf.fitfZ.conv	Bonf	f1conv	Not necessary	7
truncfdr	fdrthres	pwr*exp_density	None	1
truncfdr.corr	fdrthres	pwr*exp_density	Avg of Ghosh 1&2	2
truncfdr.corr1	fdrthres	pwr*exp_density	Ghosh1	3
truncfdr.corr2	fdrthres	pwr*exp_density	Ghosh2	4
truncfdr.corr.median	fdrthres	pwr*exp_density	Median (Zhong)	5
truncfdr.corr.MSEmedian	fdrthres	pwr*exp_density	MSE median (Zhong)	6
truncfdr.fitfZ.conv	fdrthres	f1conv	Not necessary	7

Bonf, Bonferroni correction; fdrthres, local fdr threshold; pwr*exp_density, fitting by the power times exponential density curve; flconv, fitting by considering the convolution density of non-null observations f_1_(*z*); Avg, average. The winner's curse correction methods are named by the first author of the corresponding reference papers. Please see the text for details.

### Dealing with continuous traits

Although our previous discussions focus on variance explained of binary outcomes, the method can easily be extended to continuous traits. The only difference lies in the conversion of Vg to *z* and calculation of power given a level of Vg. In fact fewer assumptions are required for continuous traits. For example, no extra information on the prevalence of disease is required.

A given level of Vg corresponds to a specific *z*-statistic and the same Vg would always give rise to the same power. In summary, we have the following relations between *z* and Vg :
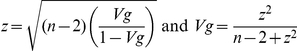
where *n* is the sample size.

Recall that we require the derivative 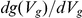 to construct the convolution density of *z*, where *g* is the function to convert Vg to *z*. In the case of a continuous outcome, one can easily obtain a closed form expression
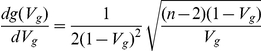
The mathematical details are given in the supplementary methods (Text S1).

### Confidence interval (CI) for *λ* and number of susceptibility variants

#### Non-parametric bootstrap

Non-parametric bootstrap can be applied to all proposed estimation methods. for construction of CIs. To perform non-parametric bootstrap, one resamples with replacement the subjects from the case-control dataset and re-calculates the test statistic for each SNP in each run. Then repeat the entire procedure of distribution fitting as described above. The *λ* found in each bootstrap run is recorded and the confidence interval of *λ* can be obtained from the empirical distribution of *λ* in bootstrap runs. The non-parametric bootstrap approach takes into account of all possible sources of uncertainties, such as uncertainties in the estimate of the odds ratios and allele frequencies and variation brought about by winner's curse correction methods. This approach is relatively straightforward and can be applied to any of the proposed estimation methods. However, the raw data on the genotypes and phenotypes of all subjects are required.

#### Parametric bootstrap

Parametric bootstrap is another way to obtain the CI when raw data is not available. The basic idea is to generate many simulated samples (each with the same of number of observations) from the known distribution of Vg or *z*-values, based on the estimated value of *λ*. Then we could re-estimate *λ* in each simulated sample and obtain a distribution for *λ*. For example, we considered fitting the pdf of the alternative *z*-values, or 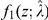. In this case, one simulates *z*-values based on the pdf 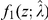. Perhaps the easiest way to simulate the *z*-values is to first derive the cdf by integrating 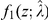 up to various points and then simulate uniform random variables. Corresponding random *z*-values can be generated from the inverse cdf.

As the bootstrap methods are more computationally intensive than analytic methods, we will focus on the latter in simulations and applications to a few real datasets. Bootstrap methods however may have better performance particularly when the number of observations is small. If access to the raw data is available, non-parametric bootstrap is also applicable and has the merit of being free from distributional assumptions.

#### Analytic approaches

A common analytic approach for calculating CI is to assume normality of the parameter and obtain the SE by inversion of the Fisher information matrix. Alternatively, one may obtain CI by inverting hypothesis tests, such as the maximum likelihood ratio test (MLRT).

These analytic methods for calculation of CI however cannot directly be applied when we estimate *λ* by fitting to the distribution of observed Vg with correction for winner's curse. Note that we assume the *actual* Vg is exponentially distributed and hence fit the power x exponential density curve to the data. What we obtain from association studies however is *not* the actual Vg but Vg with random sampling variation. Put it another way, the Vg is derived from the allele frequencies and odds ratios. Both of them have sampling variations (especially the odds ratios) and would not be identical when we repeat the study. Nevertheless our approach is to fit the estimated “true” Vg to the exponential density multiplied by power. The uncertainties of the allele frequencies and ORs have not been dealt with in the model.

Hypothetically, if one is able to fit the observed data to the actual *sampling* distribution of Vg, then the analytic approaches for obtaining CI are applicable. But since the form of this distribution is difficult to obtain, we cannot obtain CIs by analytic means directly. Correction for winner's curse introduces further variations. The effect to the variance and CI of the final *λ* estimate is not straightforward. In addition, different ways of winner's curse correction have different effects on the variances of *λ*, as shown in our simulations.

Analytic means of calculating CI can be applied when we fit the significant variants to the convolution density 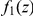 directly. Note that the sampling variation of effect size has been incorporated since the convolution already takes into account the random Gaussian noise added to the actual *z*-value.

Two standard analytic methods in deriving the confidence interval were used. The first one is based on inverting the observed Fisher information evaluated at the MLE. It may be expressed as the negative of the 2nd derivative of the log-likelihood function,
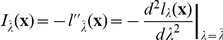
where **x** represent the observed data, *λ* is the parameter to estimate and 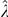 is the MLE. The confidence interval of 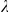 is given by
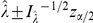
assuming normality of the parameter estimate.

The second approach is derived from the maximum likelihood ratio test (MLRT). It is based on the duality of hypothesis testing and interval estimation. To construct a confidence interval with coverage 1−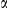, we consider the acceptance region of a test of size 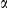 for 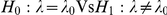. Here we just consider a single parameter for simplicity. The maximum likelihood ratio test has the following test statistic which follows a chi-square distribution:
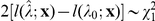
We are interested in all the lambdas such that the null hypothesis is *not* rejected, or more simply, the lambdas that are not significantly different from the MLE. The confidence interval with coverage 1−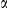 includes all 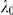 satisfying
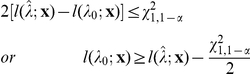



### Simulation on coverage probabilities of CIs

It is less clear whether these analytic approaches would work if we worked with weighted maximum likelihood. To assess the coverage probability of the proposed analytic CIs, we performed a brief simulation study on a sample size of 3500 cases and 3500 controls and lambdas of 1000, 2000 and 3000. One thousand simulations were carried out. In each simulation run, random *z*-values are generated as previously described. Ninety-five percent confidence intervals of the parameter *λ* were computed using two analytic methods (inversion of Fisher information matrix and MLRT), for markers passing Bonferroni correction and for those passing the local fdr threshold of 0.3. We evaluated coverage probabilities and the width of the CIs.

### Application to real data

The above distribution fitting methods were applied to a few real disease examples. As estimation of the local fdr requires all summary statistics to be available, we focused on studies that had full summary statistics released before. We included three large-scale studies, one on lipid levels [13], one on type 2 diabetes [14] and the other on Crohn's disease [15]. As the SNPs in a GWAS are correlated due to linkage disequilibrium (LD), several markers may be in strong LD with one casual variant and still show statistical significance. The mean effect size or Vg may be inflated as the signals from these proxy markers were counted as well. We experimented a rough pruning procedure to reduce the effect of correlation. The pruning was applied to the diabetes and Crohn's datasets and SNPs were pruned such that they were at least 30 kb apart.

For the Crohn's disease dataset [15], we also tried to estimate the effect sizes from the replication study alone to replace winner's curse corrections. The loci listed in Table 2 of [15] were taken to be the true associated variants. The power was calculated based on the probability of passing both the 1st-stage *p*-value threshold (<5×10^−5^) and the 2nd-stage threshold (<0.0008 or 0.05/63 by Bonferroni correction) given the effect size. *λ* was estimated by the same distribution fitting procedure as detailed previously.

**Table 2:** Root mean squared error of different estimators from simulations when number of cases and controls each equals 3500.

RMSE	*λ* = 1000		λ = 2000		λ = 3000		λ = 4000	
*N* = 3500	RMSE	rank	RMSE	rank	RMSE	rank	RMSE	rank
Bonf	198	12	783	13	1553	14	2403	11
Bonf.corr	201	13	773	12	1520	13	2841	12
Bonf.corr1	159	11	645	11	1408	11	3065	13
Bonf.corr2	237	14	845	14	1477	12	2318	10
Bonf.corr.med	123	8	443	8	1001	8	2311	9
Bonf.corr.MSEmedian	93	6	323	4	802	7	1866	7
Bonf.fitfZ.conv	91	4	352	6	1021	9	3795	14
truncfdr	140	10	625	10	1335	10	2151	8
truncfdr.corr	110	7	407	7	692	5	1035	5
truncfdr.corr1	92	5	339	5	615	4	998	4
truncfdr.corr2	126	9	446	9	694	6	936	2
truncfdr.corr.median	71	1	221	1	418	1	730	1
truncfdr.corr.MSEmedian	77	3	282	3	593	3	970	3
truncfdr.fitfZ.conv	74	2	245	2	520	2	1236	6

### Fitting other types of distributions to real data

Although we have focused on the exponential distribution in this study, we noted that this is only theoretical and may *not* exactly be the true model in real scenarios (please see the discussion section for further details). Particularly, the effect size distribution of complex traits may be more skewed towards zero than an exponential distribution, as suggested in Park et al. [6]. Therefore we have also tried to fit other types of distributions to the real data and observe how the parameter estimates will change. We have chosen to fit gamma distributions as an alternative to exponential distribution. This is because the gamma distribution is highly flexible with both shape and scale parameters. When the shape parameter is one, it is equivalent to an exponential distribution. Distributions that are more skewed towards zero than an exponential density have shape parameters less than one. We set different shape parameters and the scale parameter estimates were estimated by fitting the alternative density of *z*-values (the methodology is described under the section “Fitting the most significant z-values to the alternative density”). The mean of a gamma distribution is simply the product of the shape and scale parameters.

## Results

### Simulation results for estimators of *λ*

Tables 2, 3 and 4 shows the overall performance of 14 different estimators as measured by the root mean squared error from 300 simulations. In total 12 scenarios were studied. Firstly, we focus on the comparison of using only the most significant variants passing Bonferroni correction versus using all variants below a certain local fdr threshold. It is clear that by relaxing the *p*-value threshold and then removing the false positive effects by local fdr weighting, the root mean squared error can be reduced. The mean, bias and SD of different estimators were shown in tables S2 and S3. The variance (or SD) of the estimators decreased when we allowed more markers for fitting with adjustments for false positives using fdr. The improvement was more marked when the effect size was small (i.e. true *λ* is large) and the sample size was not large. For example when *N*_case_ = *N*_ctrl_ = 3500 and *λ* is 4000, the SD of the fdr-adjusted estimators was only about one-third of the SD of the Bonferroni- adjusted counterparts. The bias of the fdr-adjusted estimators were also in general smaller than that of the Bonferroni- adjusted ones, but for the last two (“MSEmedian” and “fitfZ.conv”[i.e. fitting 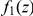]) this situation was often reversed.

**Table 3:** Root mean squared error of different estimators from simulations when number of cases and controls each equals 5000.

RMSE	*λ* = 1000		λ = 2000		λ = 3000		λ = 4000	
*N* = 5000	RMSE	rank	RMSE	rank	RMSE	rank	RMSE	rank
Bonf	131	12	570	13	1212	12	1991	14
Bonf.corr	139	13	567	12	1233	13	1874	12
Bonf.corr1	114	11	451	11	1045	11	1670	10
Bonf.corr2	161	14	656	14	1326	14	1894	13
Bonf.corr.med	103	10	348	9	746	9	1184	9
Bonf.corr.MSEmedian	84	6	243	5	532	4	959	7
Bonf.fitfZ.conv	82	5	245	6	574	6	1155	8
truncfdr	88	7	431	10	978	10	1679	11
truncfdr.corr	91	8	294	7	671	7	916	5
truncfdr.corr1	81	4	242	4	569	5	794	4
truncfdr.corr2	100	9	335	8	724	8	943	6
truncfdr.corr.median	69	2	182	1	371	1	491	1
truncfdr.corr.MSEmedian	65	1	217	3	437	3	721	3
truncfdr.fitfZ.conv	72	3	182	2	407	2	620	2

**Table 4:** Root mean squared error of different estimators from simulations when number of cases and controls each equals 7000.

RMSE	*λ* = 1000		λ = 2000		λ = 3000		λ = 4000	
*N* = 7000	RMSE	rank	RMSE	rank	RMSE	rank	RMSE	rank
Bonf	87	11	395	12	905	12	1552	12
Bonf.corr	91	13	403	13	915	13	1587	13
Bonf.corr1	78	8	319	11	733	11	1338	11
Bonf.corr2	104	14	474	14	1045	14	1715	14
Bonf.corr.med	72	5	258	9	548	8	1011	9
Bonf.corr.MSEmed	64	3	190	6	381	5	716	6
Bonf.fitfZ.conv	65	4	180	4	357	4	678	4
truncfdr	60	1	291	10	684	10	1247	10
truncfdr.corr	84	10	222	7	487	7	817	7
truncfdr.corr1	78	9	190	5	397	6	680	5
truncfdr.corr2	89	12	252	8	555	9	897	8
truncfdr.corr.median	72	6	162	3	304	3	486	3
truncfdr.corr.MSEmedian	64	2	156	1	283	2	471	2
truncfdr.fitfZ.conv	73	7	158	2	282	1	470	1

**Figure 2:**
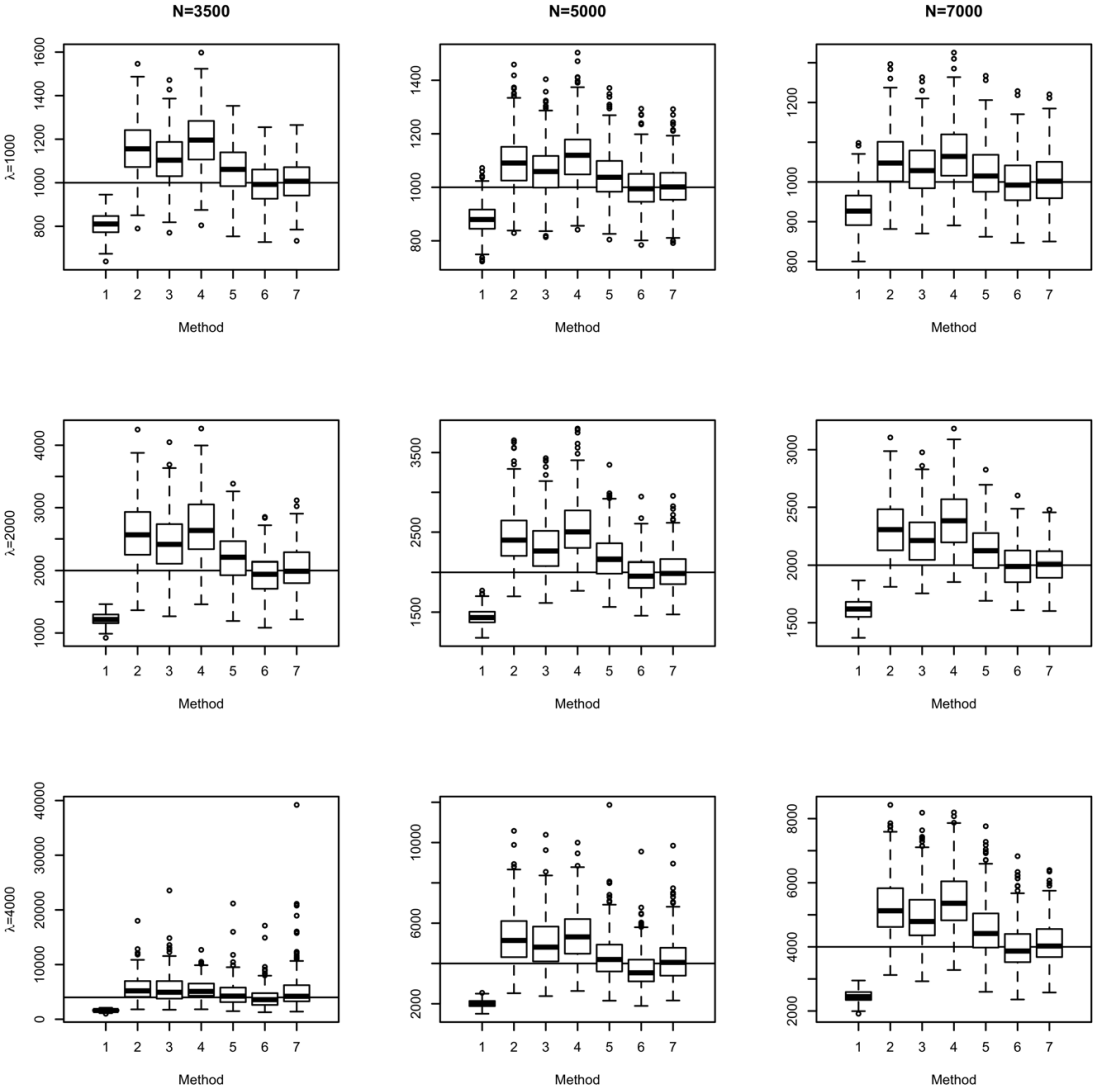
Boxplots of different estimators of *λ*, with inclusion threshold based on Bonferroni correction.

**Figure 3:**
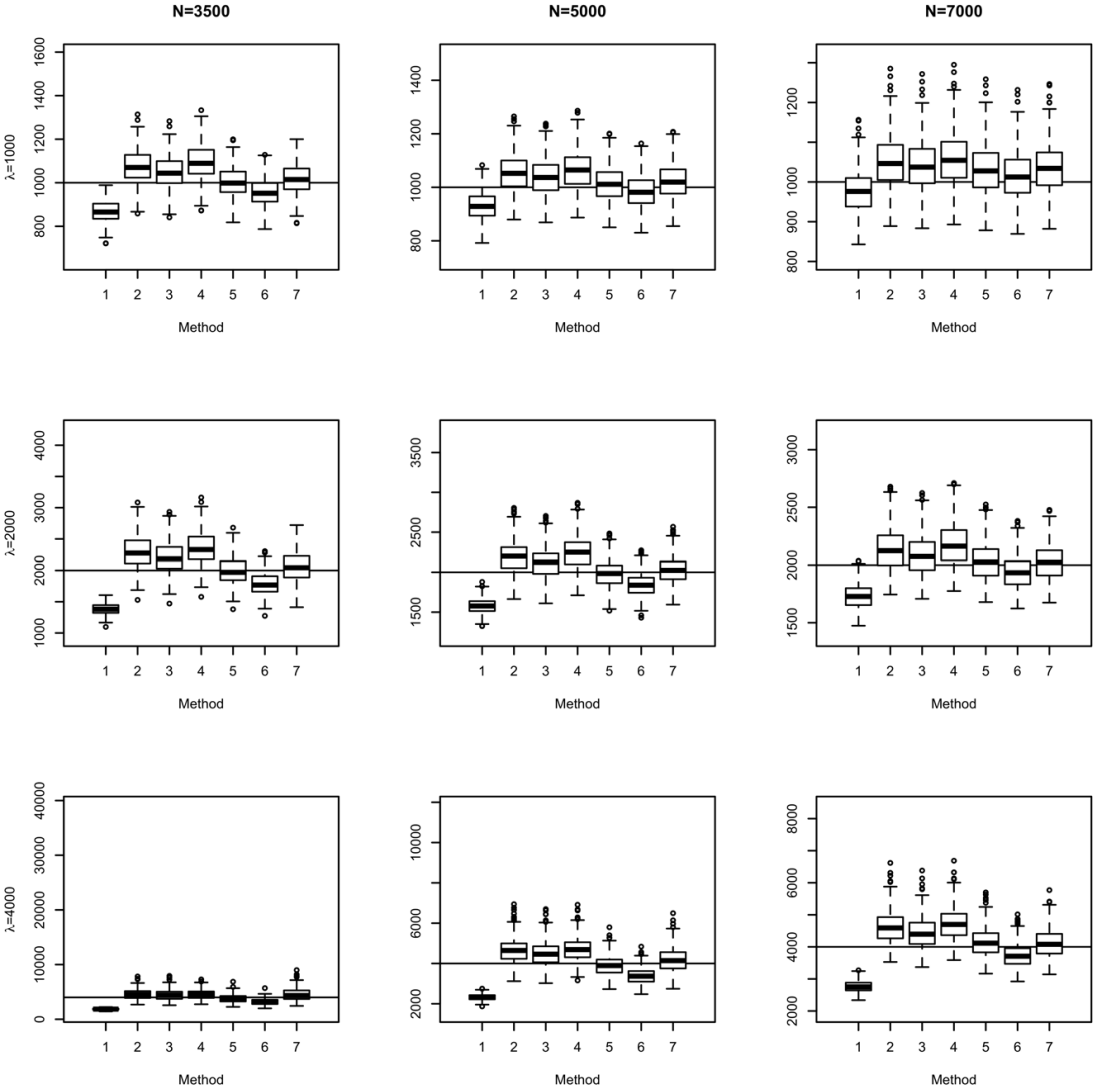
Boxplots of different estimators of *λ*, with inclusion threshold based on local fdr.

Figures 2 and 3 show the boxplots of the different estimators. For the estimators in figure 2, variants were included in fitting if they passed the Bonferroni threshold. The last two estimators (“MSEmedian” and “fitfZ.conv”) were nearly unbiased and had comparable variance. Figure 3 shows estimators that were fdr-adjusted. Overall speaking, the median and fitfZ.conv estimators had the lowest bias among all. The estimator without winner's curse correction was clearly downward biased, no matter which kind of inclusion threshold was used.

Considering the overall performance of all estimators, three estimators stood out as the best three in 10 out of 12 scenarios. They included “truncfdr.corr.median”, “truncfdr.corr.MSEmedian” and “truncfdr.fitfZ.conv” (please refer to table 1 for a description of the estimators). As expected, all three were estimators using fdr adjustment. Among winner's curse correction approaches, the median and MSE median estimators outperformed others in the current study. Fitting directly by 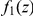 also had favorable performance.

### Simulation results for confidence intervals of *λ*

Table 5 shows the properties of CIs obtained from 1000 simulations, including coverage probabilities, mean and SD of the width of CI, and the value of the upper and lower 95% CI averaged from the simulations. Under the three simulated scenarios, all four CIs had coverage probabilities close to the theoretical value (0.95), although for “MLRT weighted” (maximum likelihood ratio test using weighted likelihood) the coverage probability seemed to be slightly lower than 0.95. By employing the weighted likelihood approach, we were able to include more variants and not surprisingly, the width of CI was much shorter than that from a standard likelihood approach. The SD of the CI width was also smaller.

**Table 5:** Simulation results for different estimates of 95% confidence intervals (CI).

		True lambda		
		1000	2000	3000
Coverage probability	Info	0.949	0.959	0.953
	Info weighted	0.936	0.955	0.969
	MLRT	0.953	0.954	0.946
	MLRT weighted	0.93	0.942	0.948
Average width of CI	Info	359	1348	3703
	Info weighted	285	877	2025
	MLRT	360	1370	4007
	MLRT weighted	285	883	2069
SD of width of CI	Info	39	296	2213
	Info weighted	24	122	454
	MLRT	39	307	2452
	MLRT weighted	25	124	477
Mean value	lowCI (info)	831	1402	1423
	upCI (info)	1190	2750	5127
	lowCI (info weighted)	882	1623	2175
	upCI (info weighted)	1166	2500	4201
	lowCI (MLRT)	843	1492	1865
	upCI (MLRT)	1203	2862	5872
	lowCI (MLRT weighted)	889	1663	2318
	upCI (MLRT weighted)	1174	2545	4387

“Info” refers to CI obtained by inversion of Fisher information matrix. “Weighted” refers to weighting by the 1-local fdr.

MLRT, maximum likelihood ratio test; lowCI, lower 95% CI; upCI, upper 95% CI.

### Results for real disease data

For Crohn's disease, we have tried an approach that did not require any winner's curse corrections. The effect sizes of the true associated variants were extracted from replication studies as described in [15]. *λ* was estimated at 1196 and hence the estimate of mean Vg was 0.0836%. The other results were summarized in table 6. Only the results based on fdr adjustment were shown since they are superior to Bonferroni-corrected estimators. Readers may refer to table S4 for the Bonferroni-corrected results for reference. The estimated values of *λ* range from about a few hundreds to around two thousand. Pruning of SNPs did not change the estimates substantially, though we noted an increase in *λ* for the Crohn's dataset after pruning. The estimated number of susceptibility variants is simply given by the heritability multiplied by *λ*. The heritabilities for type 2 DM and Crohn's disease were taken to be 0.424 [16] and 0.55 [17]. The heritabilities of HDL, LDL and TG were taken to be 0.63, 0.36 and 0.37 respectively according to Abney *et al.* [18]. As shown in table 6, the estimated number of susceptibility variants underlying the studied traits ranged from around four hundred to a thousand. Table 7 showed the estimated number of variants when we assumed a gamma distribution that allows a greater number of SNPs with smaller effects. A smaller shape parameter implies that the distribution is more skewed towards zero. As one would expect, the estimated number of variants went up as the distribution is increasingly skewed towards zero. The resulting estimates were about two to three times of the original estimate (based on exponential assumption) when the shape parameter decreased to 0.3.

**Table 6:** Estimates and confidence intervals of lambda for a few complex traits, variants included according to fdr threshold.

	HDL	LDL	TG	DM(all SNPs)	DM(pruned)	Crohn(all SNPs)	Crohn(pruned)
**Estimates of lambda**							
truncfdr	634	828	989	692	652	1086	1249
truncfdr.corr	827	1203	1534	1380	1215	1615	2062
truncfdr.corr1	813	1190	1522	1344	1150	1569	1976
truncfdr.corr2	835	1198	1519	1374	1256	1642	2117
truncfdr.corr.median	**769**	**1115**	**1403**	**1244**	**1042**	**1442**	**1787**
truncfdr.corr.MSEmedian	NA	NA	NA	1116	926	1329	1603
truncfdr.fitfZ.conv	**693**	**975**	**1192**	**1162**	**1192**	**1388**	**1715**
**Confidence intervals**							
upCI.wt (fdrthres)	751	1056	1257	1363	2420	1553	2348
loCI.wt(fdrthres)	635	895	1128	960	−36	1222	1083
upCI.MLRT.wt(fdrthres)	881	1152	1460	1380	3344	1562	2438
loCI.MLRT.wt(fdrthres)	561	835	996	975	397	1229	1183
**Esimated no. of susceptibility variants**							
based on truncfdr.corr.median	484	401	519	527	442	793	983
based on truncfdr.fitfZ.conv	437	351	441	493	505	763	943

The bolded lines refer to estimators having the best overall performance in simulations.

HDL, high density lipoprotein; LDL, low density lipoprotein; TG, triglyceride; DM, type 2 diabetes mellitus; Crohn, Crohn's disease.

Bonf, inclusion threshold based on Bonferroni correction; fdrthres, inclusion threshold set at local fdr of 0.3.

“wt” refers to weighting by 1-local fdr. Please refer to the previous tables for abbreviations of the estimators and the types of CI calculated.

**Table 7:** Estimated number of susceptibility variants assuming a gamma distribution of effect sizes.

	Shape	Lambda	Mean	Number of variants
LDL	0.9	937	9.60E-04	375
	0.7	845	8.28E-04	435
	0.5	754	6.63E-04	543
	0.3	664	4.52E-04	797
HDL	0.9	657	1.37E-03	460
	0.7	585	1.20E-03	526
	0.5	514	9.73E-04	648
	0.3	445	6.74E-04	935
TG	0.9	1152	7.81E-04	474
	0.7	1038	6.75E-04	548
	0.5	812	6.16E-04	601
	0.3	924	3.25E-04	1140
Crohn (all)	0.9	1328	6.78E-04	812
	0.7	1211	5.78E-04	951
	0.5	1093	4.57E-04	1203
	0.3	970	3.09E-04	1779
Crohn (pruned)	0.9	1649	5.46E-04	1008
	0.7	1512	4.63E-04	1188
	0.5	1375	3.64E-04	1513
	0.3	1219	2.46E-04	2235
DM (all)	0.9	1122	8.02E-04	528
	0.7	1042	6.72E-04	631
	0.5	962	5.20E-04	816
	0.3	884	3.40E-04	1249
DM (pruned)	0.9	1165	7.73E-04	549
	0.7	1095	6.39E-04	663
	0.5	1026	4.87E-04	870
	0.3	994	3.02E-04	1405

When the shape parameter equals one, the gamma distribution is equivalent to an exponential distribution and the results are listed in table 6. When the shape parameter decreases, the distribution is more skewed towards zero, implying that we assume more variants to have small effect sizes.

## Discussion

In this study we proposed a variety of methods to estimate the number of susceptibility variants in the genome based on the assumption that effect sizes are exponentially distributed.

One advantage of our methodology is that all proposed estimators require only the summary statistics (*z*-values or *p*-values) rather than the raw data, although the availability of raw data allows non-parametric bootstrap to evaluate confidence intervals. If only the *z* or *p*-values of the most significant markers are available, the models can still be fit, but one should be aware that distribution fitting on a small number of markers is often unstable.

Another feature of our methodology is that we used the variance explained as a measure of effect size. It is worth noting that the variance explained depends on both the allele frequency and the relative risk (RR). A rare variant with a large effect size may have similar Vg as a common variant with smaller effect size. For example, under a prevalence of 0.01, a variant with risk allele frequency (RAF) 0.5 and allelic RR 1.1 has the same Vg (0.0639%) as another variant with RAF 0.0005 and RR 6.348 with Vg (assuming allelic RR is multiplicative). The exponential distribution of Vg is compatible with numerous rare variants with large RR or numerous common variants with small RR, or a combination of both. Since current GWAS panels only focus on common variants, we are estimating the parameter of the exponential distribution based on the set of common SNPs, and hoping the result can be extrapolated to rare variants.

We have also applied the methodology to a number of real disease traits. The results suggested that roughly hundreds of susceptibility variants underlie these traits. This is not particularly striking, as many researchers might have expected that a large number of variants are implicated from the polygenic model for complex diseases. However, we have shown how to *quantify* the number of susceptibility variants, albeit approximately, in a statistical framework. We also noted that GWAS have been relatively successful for the traits studied here: over 20 loci have been identified for type 2 diabetes and around 30 loci have been identified for Crohn's disease [15] and lipid levels [13]. The genetic architecture of these few traits need not represent that of other complex diseases. Many diseases or traits have not been examined in large-scale or meta-analytic GWAS yet, it would be interesting to perform the analyses on these datatsets and compare the estimated number of variants for different outcomes.

### Limitations

To accurately infer the number of total susceptibility variants in the genome from GWAS data is a very difficult problem, and the methodology presented here should only be interpreted as a rough estimate based on the exponential distribution assumption. We stress that the work presented here is not a perfect solution to the problem. Instead, we made our best attempt to estimate the number of variants, and more importantly, we hoped to provide a useful and rigorous statistical framework to attack the problem.

One difficulty in dealing with GWAS data is that the SNPs are correlated, while most distribution fitting methods in statistics apply to independent observations. Often clusters of SNPs may show high significance but may just contain one or few true independent signals. As a result of the “redundant” significant signals, the mean Vg may be overestimated and *λ* underestimated. In addition the strong correlation may distort the assumed exponential distribution of effect sizes. One way to alleviate this problem is to prune the SNPs before analyses so that they are roughly independent. On the other hand, pruning reduces the number of markers available for distribution fitting and will increase the variability of the estimates, especially when the effect sizes are small and there are few markers considered significant even in the entire set of genotyped SNPs. There is no perfect solution to this sort of bias-variance tradeoff. However, as the sample size becomes larger, more SNPs will pass the significance threshold and there will be adequate SNPs for stable parameter estimates even if the SNPs are pruned prior to analysis.

Also, the significant associations in a GWAS may not be causal variants themselves, but maybe merely tagging them. We have to assume that the variance explained by significant results in a GWAS is close to that of the causal variants if they were typed.

Current GWAS technologies mainly aim at capturing common variants. As a result, the best thing we can do is to infer the total number of underlying based on the effect sizes of common variations. The contribution of rare variants and structural variations to complex diseases is largely unexplored in the literature. In addition, gene-gene or gene-environmental interactions may also play a role in the etiology of complex diseases, but our current understanding regarding interactions is still very limited. To make predictions on the total number of susceptibility variants in the genome, inevitably we have made the assumption that the other variants that are not captured share a similar distribution of Vg as common variants. Of course this assumption may not work very well in practice. Nonetheless, we think this limitation may become less of a concern in the future when studies accumulate and technologies improve. While now we can only fit the Vg of common variants, with more rare and structural variants discovered in the future, their effect sizes may also be fit using the proposed framework. Interactions may be regarded as multi-locus genotypes and their Vg can be readily calculated and distribution fitting can be done as well. The framework we presented does not pose any restrictions on the actual nature of the susceptibility variants. With accumulation of more studies we will hopefully be able to obtain more reliable estimates considering a greater variety of genetic variations.

We assumed an exponential distribution of Vg in this study, which is justified by theories in adaptation [3]. The exponential distribution is simple with only one parameter, hence the estimation can be quite reliable despite modest sample size. Employing more flexible models with more parameters would allow better fitting when the true distribution is not exponential. However, we will need much larger sample sizes such that there are enough variants passing the significance threshold (Bonferroni or fdr) that are available for fitting. Otherwise, the estimate will be unstable.

Despite the advantages, we must point out that the exponential assumption is a theoretical prediction after all, and may *not* be exactly true in practice. For example, Park et al.[6] suggested recently that the effect size distributions for complex traits may be more skewed to the left than an exponential distribution. As a result, we may have underestimated the total number of susceptibility loci. In view of this limitation, we have also tried to fit gamma distributions with shape parameters less than one, which allow more variants with small effect sizes than an exponential distribution. When the shape parameter dropped to 0.3, the estimated number of loci increased by about 2 to 3 times compared to the case when the exponential distribution is fitted. The new estimates are mostly in the range of one to two thousands. This gives a rough idea of the range of estimates if the distribution of effect size is more left-skewed.

As first mentioned in the introduction, Park et al. [6] recently have worked on a similar problem. It is notable that Park et al. have proposed a way to deal with the problem in a non-parametric manner without relying on estimate of total heritability. However, it should be noted that we are considering the *entire* range of effect sizes rather than the observed range, which is a bit trickier. The unobserved effect sizes are likely to be smaller than the observed ones and cannot be modeled easily. Note that non-parametric methods are usually highly flexible. They can fit the observed data-points well (and may perform well within the range of effect sizes observed). However, flexible models also increase the risk of overfitting. They may be less reliable at predicting future data-points as they may magnify the “noise” or random fluctuations in the original data. The danger of overfitting is exaggerated when we have to extrapolate *beyond* the observed spectrum of effect sizes. The risk of overfitting is also heightened when the number of loci (i.e. data-points) available for fitting is small in the first place, as explained earlier.

As a result, we have focused on the more restrictive parametric models in the current study, and in particular the exponential model. This is not because the exponential model is perfect, but it is the only parametric model with theoretical support and represents the best “educated guess” of effect size distribution in our view. One may change the exponential assumption to other models such as gamma distributions, as was done in this study. With greater sample sizes for association studies and more loci available for fitting, one may be able to reliably fit statistical distributions with more free parameters, for example the gamma distribution (which is very flexible) with free shape and rate parameters. The reliance on the exponential distribution can then be relaxed. The framework presented in this paper can potentially be extended to deal with other types of distributions.

It should be noted that our methodology requires an external estimate of the total heritability. As there are many different statistical approaches and designs that are available for heritability studies, one may need to be aware of the limitations of different heritability estimates. For example, twin and adoption studies can separate shared environmental from shared genetic factors in families, while family-based studies (e.g. those based on siblings and parent-offsprings only) cannot make a distinction between the two types of shared effects.

We have proposed an fdr-based approach to relax the significance threshold so that more markers are available for fitting. Simulations showed that this approach had superior performance when compared to the conventional way of including only the markers that passed the Bonferroni threshold. In practice, confounding factors such as population stratification may be present to increase the false positive rate. Many methods such as principal component analysis are available for correcting population stratification [19]. If major residual confounding is suspected, for example the genomic inflation factor (*λ*) [20] remains high after correction by principal component analysis, then one should be cautious in relaxing the significance threshold. In such cases, one should also be alert to the most significant markers being false positives, and extensive replications are necessary to confirm the results. One may also consider correcting the results by genomic control [20].

Goldstein [21] presented an alternative approach to estimate the number of variants underlying height in a recent commentary. His approach is different from ours and Park et al. [6]. In [6] and our study, the authors considered the probability density function of effect sizes and obtained parameters estimates by distribution fitting. Goldstein considered the entire range of effect sizes as we do. However, he ranked the SNP from largest to smallest effect sizes and plotted the effect size (in variance explained) (on y-axis) against the rank of each SNP (on x-axis). An exponential function curve was fit to the observed data-points with least-squares regression. Essentially the rank of SNP is the predictor variable and the effect size is the outcome variable. The number of variants is solved using integration. This is not the same as the probably more intuitive and conventional distribution fitting methods in which one fits a distribution to the histogram of effect sizes (i.e. y-axis is the density, x-axis is the effect size). Goldstein's method was only described briefly and the statistical methodology itself was not the major focus of the commentary. No detailed rationale for the approach or simulations was presented. Hence we are unable to make rigorous and comprehensive comparisons of our approach to that described in Goldstein. However, it is clear that in Goldstein's approach, the power of study is *not* considered and as admitted by the author, it is assumed that all SNPs yet to be found have smaller effects than the weakest one discovered to date. This will lead to an overestimation of the number of variants.

Estimation of the number of variants underlying a trait is a very challenging task. We believe that for most complex diseases, the Vg of susceptibility variants are likely to be rather small. It may be possible to investigate the aggregate effect of these variants, for example Purcell *et al.* [22] showed a large number of SNPs (with *p* values up to 0.5) from a schizophrenia GWAS demonstrate predictive power collectively. It will be much harder to estimate the *number* of variants making up this combined effect as the Vg of *each* variant is probably tiny. For instance, it may be difficult to distinguish between 100 variants having an average Vg of 0.01% versus 10 variants having an average Vg of 0.1%.

In conclusion, we have developed a novel statistical approach to estimate the number of susceptibility variants in the genome. The performance of different proposed estimators were tested by simulations and applied to some real data examples. Despite the limitations, we believe this study is a useful step towards the understanding of the genetic architecture of complex diseases.

## Supporting Information

Text S1 Supplementary methods: Equivalence of the maximum likelihood and method of moments estimates of lambda and the approach to continuous traits.
(0.10 MB PDF)

Table S1 Power estimates for different risk allele frequencies under the same Vg.
(0.15 MB DOC)

Table S2 Mean and bias of different estimators from simulations.
(0.11 MB DOC)

Table S3 SD of different estimators from simulations.
(0.07 MB DOC)

Table S4 Estimates and confidence intervals of lambda for a few complex traits, with inclusion threshold based on Bonferroni correction.
(0.04 MB DOC)
