# Supplementary material for: Estimating the Total Number of Susceptibility Variants Underlying Complex Diseases from Genome-Wide Association Studies
Source: PLoS One. 2010 Nov 17;5(11):e13898. doi: 10.1371/journal.pone.0013898 (PMC2984437; doi:10.1371/journal.pone.0013898)
Supplement: Text S1 — Supplementary methods: Equivalence of the maximum likelihood and method of moments estimates of lambda and the approach to continuous traits. (0.10 MB PDF) [file pone.0013898.s001.pdf]

Supplementary methods for

Estimating the total number of susceptibility variants underlying complex diseases from genome-wide association studies

by Hon-Cheong So, Benjamin H.K. Yip and Pak C. Sham

1. Maximum likelihood estimate of λ and equivalence to method of moments estimate

For notational simplicity, we use x to denote variance explained and ϕ(x) to denote the power function for x.

The density function of x is

$$f(x) = \frac{\lambda e^{-\lambda x}\phi(x)}{\int_0^1 \lambda e^{-\lambda x}\phi(x)dx}$$

The likelihood is given by

$$L(\lambda) = \prod_{i=1}^{n} \frac{\lambda e^{-\lambda x_i}\phi(x_i)}{\int_0^1 \lambda e^{-\lambda x}\phi(x)dx}$$

and the log-likelihood is

$$\begin{aligned} l(\lambda) &= \ln L(\lambda) \\ &= \sum_{i=1}^{n} \left\{ \ln \lambda - \lambda x_i + \ln[\phi(x_i)] - \ln \int_0^1 \lambda e^{-\lambda x}\phi(x)dx \right\} \\ &= n \ln \lambda - \lambda \sum_{i=1}^{n} x_i + \sum_{i=1}^{n} \ln[\phi(x_i)] - n \ln \int_0^1 \lambda e^{-\lambda x}\phi(x)dx \end{aligned}$$

Differentiating with respect to λ and setting the derivative to zero yields

$$\frac{n}{\lambda}-\sum_{i=1}^{n}x_i - n\left\{\frac{d}{d\lambda}\left[\ln\int_0^1 \lambda e^{-\lambda x}\phi(x)dx\right]\right\}=0$$

$$\frac{n}{\lambda}-\sum_{i=1}^{n}x_i - n\left\{\frac{\int_0^1 \frac{\partial}{\partial\lambda}\lambda e^{-\lambda x}\phi(x)dx}{\int_0^1 \lambda e^{-\lambda x}\phi(x)dx}\right\}=0$$

$$\frac{n}{\lambda}-\sum_{i=1}^{n}x_i - n\left\{\frac{\int_0^1 (-x\lambda e^{-\lambda x}+e^{-\lambda x})\phi(x)dx}{\int_0^1 \lambda e^{-\lambda x}\phi(x)dx}\right\}=0$$

$$\frac{n}{\lambda}-\sum_{i=1}^{n}x_i + \frac{n\int_0^1 x\lambda e^{-\lambda x}\phi(x)dx}{\int_0^1 \lambda e^{-\lambda x}\phi(x)dx} - \frac{n\int_0^1 e^{-\lambda x}\phi(x)dx}{\lambda\int_0^1 e^{-\lambda x}\phi(x)dx}=0$$

$$\frac{\int_0^1 x\lambda e^{-\lambda x}\phi(x)dx}{\int_0^1 \lambda e^{-\lambda x}\phi(x)dx}=\frac{\sum_{i=1}^{n}x_i}{n}$$

which is equivalent to matching the population mean with the sample mean. Thus the estimation of λ by both maximum likelihood and the method of moments require solving exactly the same equation.

In a similar vein, we can prove that matching the theoretical mean with the weighted sample mean (in this case weights are derived from local fdr) is equivalent to performing weighted ML fitting. The weighted likelihood is given by

$$\sum_i w_i \ln L_i(\lambda)$$

$$=\sum_i w_i \ln\lambda - \lambda\sum_i w_i x_i + \sum_i w_i \ln[\phi(x_i)] - \sum_i w_i \ln\int_0^1 \lambda e^{-\lambda x}\phi(x)dx$$

Differentiating with respect to λ and setting the derivative to zero gives

$$\frac{d}{d\lambda}\sum_i w_i \ln L_i(\lambda)=0$$

$$\frac{1}{\lambda}\sum_i w_i - \sum_i w_i x_i - \sum_i w_i \frac{d}{d\lambda}\left\{\ln\int_0^1 \lambda e^{-\lambda x}\phi(x)dx\right\}=0$$

$$\frac{1}{\lambda}\sum_i w_i - \sum_i w_i x_i - \sum_i w_i \left\{\frac{-\int_0^1 x\lambda e^{-\lambda x}\phi(x)dx + \int_0^1 e^{-\lambda x}\phi(x)dx}{\int_0^1 \lambda e^{-\lambda x}\phi(x)dx}\right\}=0$$

$$\frac{\int_0^1 x\lambda e^{-\lambda x}\phi(x)dx}{\int_0^1 \lambda e^{-\lambda x}\phi(x)dx}=\frac{\sum_i w_i x_i}{\sum_i w_i}$$

2. Dealing with continuous traits

For a linear regression of the form $Y = \beta_0 + \beta_1 x$ and estimated regression coefficients b_0 and b_1, we have the following ANOVA table [20]:

ANOVA table for linear regression

	Sum of squares	Df	Mean sum of squares
Regression	$SSR = \sum(\hat{Y}_i - \bar{Y})^2$	1	$MSR = SSR / 1$
Error	$SSE = \sum(Y_i - \hat{Y}_i)^2$	n-2	$MSE = SSE / (n-2)$
Total	$SSTO = \sum(Y_i - \bar{Y})^2$	n-1	

where $\hat{Y}_i$ is the fitted value for the i th observation, $\bar{Y}$ is the mean value of the outcome variable and n is the sample size.

The F statistic is given by

$$F = \frac{MSR}{MSE} = \frac{SSR}{SSE / (n-2)} = \frac{b_1^2 \sum(X_i - \bar{X})}{MSE}$$

The variance of b_1 is

$$\text{var}(b_1) = \frac{MSE}{\sum(X_i - \bar{X})}$$

Hence the F statistic equal to the square of the t-statistic:

$$F = \left(\frac{b_1}{se(b_1)}\right)^2 = t^2$$

On the other hand, the F statistic is directly related to Vg:

$$Vg = \frac{SSR}{SSTO}$$

$$SSR = SSTO \times Vg$$

Since

$$SSE + SSR = SSTO$$

$$SSE = SSTO - SSTO \times Vg = SSTO(1 - Vg)$$

thus we have

$$F = \frac{Vg}{(1 - Vg)/(n-2)}$$

For large sample size *n*, the t distribution is very close to the normal distribution, thus the following approximation can be made

$$F = \frac{Vg}{(1 - Vg)/(n-2)} = t^2 \approx z^2$$

Hence a given level of Vg corresponds to a specific *z*-statistic and the same Vg would always give rise to the same power. In summary, we have the following relations between *z* and Vg :

$$z = \sqrt{(n-2)\left(\frac{Vg}{1-Vg}\right)} \quad \text{and} \quad Vg = \frac{z^2}{n-2+z^2}$$

Recall that we require the derivative $dg(V_g)/dV$ to construct the convolution density of *z*, where *g* is the function to convert Vg to *z*. In the case of a continuous outcome, one can obtain a closed form expression

$$\frac{dg(V_g)}{dV_g} = \frac{d}{dV_g}\sqrt{(n-2)\left(\frac{V_g}{1-V_g}\right)}$$

$$= \left(\sqrt{n-2}\right)\frac{1}{2}\left(\frac{V_g}{1-V_g}\right)^{-\frac{1}{2}}\frac{d}{dV_g}\left(\frac{V_g}{1-V_g}\right)$$

$$= \frac{1}{2(1-V_g)^2}\sqrt{\frac{(n-2)(1-V_g)}{V_g}}$$
