# Supplementary material for: Estimating the Total Number of Susceptibility Variants Underlying Complex Diseases from Genome-Wide Association Studies
Source: PLoS One. 2010 Nov 17;5(11):e13898. doi: 10.1371/journal.pone.0013898 (PMC2984437; doi:10.1371/journal.pone.0013898)
Supplement: Table S1 — Power estimates for different risk allele frequencies under the same Vg. (0.15 MB DOC) [file pone.0013898.s002.doc]

Table S1 Power estimates for different risk allele frequencies under the same Vg.

|  |  |  |  |  |  | Variance explained | |  |  |  |  |
| --- | --- | --- | --- | --- | --- | --- | --- | --- | --- | --- | --- |
|  | Vg=0.001 | 0.0015 | 0.002 | 0.0025 | 0.003 | 0.0035 | 0.004 | 0.0045 | 0.005 | 0.0055 | 0.006 |
| alpha=5e-5, N=3500 | |  |  |  |  |  |  |  |  |  |  |
| Freq of risk allele |  |  |  |  |  |  |  |  |  |  |  |
| 0.1 | 0.075 | 0.200 | 0.368 | 0.544 | 0.697 | 0.813 | 0.893 | 0.942 | 0.970 | 0.985 | 0.993 |
| 0.15 | 0.074 | 0.196 | 0.362 | 0.536 | 0.689 | 0.806 | 0.887 | 0.938 | 0.967 | 0.984 | 0.992 |
| 0.2 | 0.073 | 0.194 | 0.358 | 0.530 | 0.683 | 0.801 | 0.883 | 0.935 | 0.965 | 0.983 | 0.992 |
| 0.25 | 0.072 | 0.192 | 0.354 | 0.526 | 0.678 | 0.797 | 0.879 | 0.932 | 0.964 | 0.982 | 0.991 |
| 0.3 | 0.072 | 0.190 | 0.351 | 0.522 | 0.674 | 0.793 | 0.876 | 0.930 | 0.962 | 0.981 | 0.990 |
| 0.35 | 0.071 | 0.189 | 0.349 | 0.519 | 0.671 | 0.790 | 0.874 | 0.928 | 0.961 | 0.980 | 0.990 |
| 0.4 | 0.071 | 0.188 | 0.347 | 0.516 | 0.668 | 0.787 | 0.871 | 0.926 | 0.960 | 0.979 | 0.989 |
| 0.45 | 0.070 | 0.187 | 0.345 | 0.513 | 0.664 | 0.784 | 0.869 | 0.924 | 0.959 | 0.978 | 0.989 |
| 0.5 | 0.070 | 0.185 | 0.342 | 0.510 | 0.661 | 0.781 | 0.866 | 0.923 | 0.957 | 0.977 | 0.988 |
| 0.55 | 0.070 | 0.184 | 0.340 | 0.507 | 0.658 | 0.778 | 0.864 | 0.921 | 0.956 | 0.976 | 0.988 |
| 0.6 | 0.069 | 0.183 | 0.338 | 0.504 | 0.654 | 0.774 | 0.861 | 0.918 | 0.954 | 0.975 | 0.987 |
| 0.65 | 0.069 | 0.182 | 0.336 | 0.501 | 0.651 | 0.771 | 0.858 | 0.916 | 0.953 | 0.974 | 0.987 |
| 0.7 | 0.068 | 0.180 | 0.333 | 0.497 | 0.646 | 0.767 | 0.854 | 0.913 | 0.951 | 0.973 | 0.986 |
| 0.75 | 0.068 | 0.178 | 0.330 | 0.493 | 0.641 | 0.762 | 0.850 | 0.910 | 0.948 | 0.971 | 0.985 |
| 0.8 | 0.067 | 0.176 | 0.326 | 0.487 | 0.635 | 0.755 | 0.845 | 0.906 | 0.945 | 0.969 | 0.983 |
| 0.85 | 0.066 | 0.173 | 0.320 | 0.479 | 0.626 | 0.747 | 0.837 | 0.899 | 0.940 | 0.966 | 0.981 |
|  |  |  |  |  |  |  |  |  |  |  |  |
| alpha=1e-5, N=3500 | |  |  |  |  |  |  |  |  |  |  |
| Freq of risk allele |  |  |  |  |  |  |  |  |  |  |  |
| 0.1 | 0.036 | 0.114 | 0.242 | 0.401 | 0.562 | 0.702 | 0.810 | 0.886 | 0.936 | 0.965 | 0.982 |
| 0.15 | 0.035 | 0.112 | 0.237 | 0.393 | 0.552 | 0.692 | 0.802 | 0.880 | 0.931 | 0.962 | 0.980 |
| 0.2 | 0.035 | 0.110 | 0.234 | 0.388 | 0.546 | 0.685 | 0.796 | 0.875 | 0.927 | 0.960 | 0.979 |
| 0.25 | 0.034 | 0.109 | 0.231 | 0.384 | 0.540 | 0.680 | 0.791 | 0.871 | 0.924 | 0.958 | 0.977 |
| 0.3 | 0.034 | 0.108 | 0.229 | 0.380 | 0.536 | 0.676 | 0.787 | 0.868 | 0.922 | 0.956 | 0.976 |
| 0.35 | 0.034 | 0.107 | 0.227 | 0.377 | 0.532 | 0.671 | 0.783 | 0.864 | 0.920 | 0.954 | 0.975 |
| 0.4 | 0.034 | 0.106 | 0.225 | 0.374 | 0.528 | 0.668 | 0.779 | 0.861 | 0.917 | 0.953 | 0.974 |
| 0.45 | 0.033 | 0.105 | 0.223 | 0.371 | 0.525 | 0.664 | 0.776 | 0.859 | 0.915 | 0.951 | 0.973 |
| 0.5 | 0.033 | 0.104 | 0.221 | 0.368 | 0.522 | 0.660 | 0.772 | 0.856 | 0.913 | 0.950 | 0.972 |
| 0.55 | 0.033 | 0.104 | 0.220 | 0.366 | 0.518 | 0.656 | 0.769 | 0.853 | 0.910 | 0.948 | 0.971 |
| 0.6 | 0.033 | 0.103 | 0.218 | 0.363 | 0.514 | 0.652 | 0.765 | 0.849 | 0.908 | 0.946 | 0.970 |
| 0.65 | 0.032 | 0.102 | 0.216 | 0.360 | 0.510 | 0.648 | 0.761 | 0.846 | 0.905 | 0.944 | 0.968 |
| 0.7 | 0.032 | 0.101 | 0.214 | 0.356 | 0.506 | 0.643 | 0.756 | 0.841 | 0.902 | 0.941 | 0.966 |
| 0.75 | 0.032 | 0.100 | 0.211 | 0.352 | 0.500 | 0.637 | 0.750 | 0.836 | 0.897 | 0.938 | 0.964 |
| 0.8 | 0.031 | 0.098 | 0.208 | 0.347 | 0.493 | 0.629 | 0.743 | 0.830 | 0.892 | 0.934 | 0.961 |
| 0.85 | 0.031 | 0.096 | 0.204 | 0.340 | 0.484 | 0.619 | 0.732 | 0.820 | 0.884 | 0.928 | 0.957 |
|  |  |  |  |  |  |  |  |  |  |  |  |
| alpha=5e-5, N=5000 | |  |  |  |  |  |  |  |  |  |  |
| 0.1 | 0.176 | 0.415 | 0.651 | 0.822 | 0.921 | 0.968 | 0.989 | 0.996 | 0.9988 | 0.9997 | 0.9999 |
| 0.15 | 0.174 | 0.409 | 0.644 | 0.816 | 0.916 | 0.966 | 0.987 | 0.996 | 0.9986 | 0.9996 | 0.9999 |
| 0.2 | 0.172 | 0.405 | 0.639 | 0.811 | 0.913 | 0.964 | 0.987 | 0.995 | 0.9985 | 0.9995 | 0.9999 |
| 0.25 | 0.170 | 0.402 | 0.635 | 0.808 | 0.911 | 0.963 | 0.986 | 0.995 | 0.9984 | 0.9995 | 0.9999 |
| 0.3 | 0.169 | 0.399 | 0.632 | 0.805 | 0.909 | 0.961 | 0.985 | 0.995 | 0.9982 | 0.9995 | 0.9998 |
| 0.35 | 0.168 | 0.397 | 0.629 | 0.802 | 0.907 | 0.960 | 0.985 | 0.994 | 0.9981 | 0.9994 | 0.9998 |
| 0.4 | 0.167 | 0.394 | 0.626 | 0.799 | 0.905 | 0.959 | 0.984 | 0.994 | 0.9980 | 0.9994 | 0.9998 |
| 0.45 | 0.166 | 0.392 | 0.623 | 0.797 | 0.903 | 0.958 | 0.983 | 0.994 | 0.9979 | 0.9993 | 0.9998 |
| 0.5 | 0.166 | 0.390 | 0.620 | 0.795 | 0.901 | 0.957 | 0.983 | 0.994 | 0.9978 | 0.9993 | 0.9998 |
| 0.55 | 0.165 | 0.388 | 0.618 | 0.792 | 0.899 | 0.956 | 0.982 | 0.993 | 0.9977 | 0.9992 | 0.9998 |
| 0.6 | 0.164 | 0.386 | 0.615 | 0.789 | 0.897 | 0.955 | 0.982 | 0.993 | 0.9975 | 0.9992 | 0.9997 |
| 0.65 | 0.163 | 0.384 | 0.612 | 0.786 | 0.895 | 0.953 | 0.981 | 0.993 | 0.9974 | 0.9991 | 0.9997 |
| 0.7 | 0.162 | 0.382 | 0.608 | 0.783 | 0.893 | 0.952 | 0.980 | 0.992 | 0.9972 | 0.9990 | 0.9997 |
| 0.75 | 0.161 | 0.379 | 0.604 | 0.779 | 0.890 | 0.950 | 0.979 | 0.992 | 0.9969 | 0.9989 | 0.9996 |
| 0.8 | 0.159 | 0.375 | 0.599 | 0.774 | 0.886 | 0.947 | 0.977 | 0.991 | 0.9966 | 0.9988 | 0.9996 |
| 0.85 | 0.157 | 0.370 | 0.592 | 0.767 | 0.880 | 0.944 | 0.975 | 0.990 | 0.9960 | 0.9985 | 0.9995 |
|  |  |  |  |  |  |  |  |  |  |  |  |
| alpha=1e-5, N=5000 | |  |  |  |  |  |  |  |  |  |  |
| 0.1 | 0.098 | 0.282 | 0.511 | 0.713 | 0.853 | 0.933 | 0.972 | 0.989 | 0.9963 | 0.9988 | 0.9996 |
| 0.15 | 0.097 | 0.277 | 0.503 | 0.705 | 0.846 | 0.928 | 0.970 | 0.988 | 0.9958 | 0.9986 | 0.9996 |
| 0.2 | 0.095 | 0.273 | 0.498 | 0.699 | 0.841 | 0.925 | 0.968 | 0.987 | 0.9954 | 0.9984 | 0.9995 |
| 0.25 | 0.094 | 0.271 | 0.493 | 0.694 | 0.837 | 0.922 | 0.966 | 0.987 | 0.9950 | 0.9983 | 0.9994 |
| 0.3 | 0.094 | 0.268 | 0.490 | 0.690 | 0.834 | 0.920 | 0.965 | 0.986 | 0.9947 | 0.9981 | 0.9994 |
| 0.35 | 0.093 | 0.266 | 0.487 | 0.687 | 0.831 | 0.918 | 0.964 | 0.985 | 0.9944 | 0.9980 | 0.9993 |
| 0.4 | 0.092 | 0.265 | 0.484 | 0.684 | 0.828 | 0.916 | 0.963 | 0.985 | 0.9941 | 0.9979 | 0.9993 |
| 0.45 | 0.092 | 0.263 | 0.481 | 0.681 | 0.826 | 0.914 | 0.962 | 0.984 | 0.9938 | 0.9978 | 0.9992 |
| 0.5 | 0.091 | 0.261 | 0.478 | 0.677 | 0.823 | 0.912 | 0.960 | 0.983 | 0.9935 | 0.9976 | 0.9992 |
| 0.55 | 0.091 | 0.259 | 0.475 | 0.674 | 0.820 | 0.910 | 0.959 | 0.983 | 0.9932 | 0.9975 | 0.9991 |
| 0.6 | 0.090 | 0.258 | 0.472 | 0.671 | 0.817 | 0.908 | 0.958 | 0.982 | 0.9928 | 0.9973 | 0.9990 |
| 0.65 | 0.089 | 0.256 | 0.469 | 0.667 | 0.814 | 0.906 | 0.956 | 0.981 | 0.9924 | 0.9971 | 0.9990 |
| 0.7 | 0.089 | 0.254 | 0.466 | 0.663 | 0.810 | 0.903 | 0.955 | 0.980 | 0.9919 | 0.9969 | 0.9989 |
| 0.75 | 0.088 | 0.251 | 0.461 | 0.658 | 0.806 | 0.900 | 0.952 | 0.979 | 0.9913 | 0.9966 | 0.9987 |
| 0.8 | 0.087 | 0.248 | 0.456 | 0.652 | 0.800 | 0.896 | 0.950 | 0.977 | 0.9904 | 0.9961 | 0.9985 |
| 0.85 | 0.085 | 0.244 | 0.449 | 0.644 | 0.792 | 0.889 | 0.946 | 0.975 | 0.9891 | 0.9955 | 0.9982 |

Alpha, type I error threshold. N, sample size (e.g. N=3500 denotes 3500 cases and 3500 controls)
