# Supplementary material for: Estimating the Total Number of Susceptibility Variants Underlying Complex Diseases from Genome-Wide Association Studies
Source: PLoS One. 2010 Nov 17;5(11):e13898. doi: 10.1371/journal.pone.0013898 (PMC2984437; doi:10.1371/journal.pone.0013898)
Supplement: Table S2 — Mean and bias of different estimators from simulations. (0.11 MB DOC) [file pone.0013898.s003.doc]

Table S2 Mean and bias of different estimators from simulations

|  | *λ*=1000 |  | λ=2000 |  | λ=3000 |  | λ=4000 |  |
| --- | --- | --- | --- | --- | --- | --- | --- | --- |
| *N*=3000 | Mean | bias | Mean | bias | Mean | bias | Mean | bias |
| Bonf | 809 | -191 | 1223 | -777 | 1453 | -1547 | 1605 | -2395 |
| Bonf.corr | 1158 | 158 | 2606 | 606 | 4052 | 1052 | 5689 | 1689 |
| Bonf.corr1 | 1109 | 109 | 2452 | 452 | 3853 | 853 | 5606 | 1606 |
| Bonf.corr2 | 1197 | 197 | 2696 | 696 | 4093 | 1093 | 5461 | 1461 |
| Bonf.corr.med | 1061 | 61 | 2216 | 216 | 3342 | 342 | 4698 | 698 |
| Bonf.corr.MSEmedian | 992 | -8 | 1943 | -57 | 2836 | -164 | 3937 | -63 |
| Bonf.fitfZ.conv | 1006 | 6 | 2048 | 48 | 3251 | 251 | 5262 | 1262 |
| truncfdr | 868 | -132 | 1381 | -619 | 1669 | -1331 | 1853 | -2147 |
| truncfdr.corr | 1075 | 75 | 2297 | 297 | 3467 | 467 | 4568 | 568 |
| truncfdr.corr1 | 1048 | 48 | 2207 | 207 | 3338 | 338 | 4442 | 442 |
| truncfdr.corr2 | 1095 | 95 | 2350 | 350 | 3502 | 502 | 4523 | 523 |
| truncfdr.corr.median | 1004 | 4 | 1994 | -6 | 2909 | -91 | 3802 | -198 |
| truncfdr.corr.MSEmedian | 956 | -44 | 1782 | -218 | 2504 | -496 | 3201 | -799 |
| truncfdr.fitfZ.conv | 1019 | 19 | 2057 | 57 | 3146 | 146 | 4528 | 528 |
|  |  |  |  |  |  |  |  |  |
|  |  |  |  |  |  |  |  |  |
| *N*=5000 |  |  |  |  |  |  |  |  |
| Bonf | 882 | -118 | 1439 | -561 | 1796 | -1204 | 2017 | -1983 |
| Bonf.corr | 1095 | 95 | 2444 | 444 | 3956 | 956 | 5332 | 1332 |
| Bonf.corr1 | 1063 | 63 | 2308 | 308 | 3720 | 720 | 5042 | 1042 |
| Bonf.corr2 | 1121 | 121 | 2545 | 545 | 4089 | 1089 | 5433 | 1433 |
| Bonf.corr.med | 1045 | 45 | 2190 | 190 | 3386 | 386 | 4361 | 361 |
| Bonf.corr.MSEmedian | 1002 | 2 | 1981 | -19 | 2938 | -62 | 3690 | -310 |
| Bonf.fitfZ.conv | 1007 | 7 | 2015 | 15 | 3108 | 108 | 4228 | 228 |
| truncfdr | 931 | -69 | 1580 | -420 | 2032 | -968 | 2327 | -1673 |
| truncfdr.corr | 1055 | 55 | 2202 | 202 | 3490 | 490 | 4671 | 671 |
| truncfdr.corr1 | 1039 | 39 | 2128 | 128 | 3354 | 354 | 4492 | 492 |
| truncfdr.corr2 | 1067 | 67 | 2255 | 255 | 3565 | 565 | 4733 | 733 |
| truncfdr.corr.median | 1014 | 14 | 1994 | -6 | 3014 | 14 | 3918 | -82 |
| truncfdr.corr.MSEmedian | 986 | -14 | 1850 | -150 | 2684 | -316 | 3389 | -611 |
| truncfdr.fitfZ.conv | 1024 | 24 | 2033 | 33 | 3111 | 111 | 4196 | 196 |
|  |  |  |  |  |  |  |  |  |
|  |  |  |  |  |  |  |  |  |
| *N*=7000 |  |  |  |  |  |  |  |  |
| Bonf | 930 | -70 | 1619 | -381 | 2105 | -895 | 2459 | -1541 |
| Bonf.corr | 1055 | 55 | 2316 | 316 | 3749 | 749 | 5275 | 1275 |
| Bonf.corr1 | 1035 | 35 | 2217 | 217 | 3540 | 540 | 4968 | 968 |
| Bonf.corr2 | 1071 | 71 | 2395 | 395 | 3897 | 897 | 5449 | 1449 |
| Bonf.corr.med | 1022 | 22 | 2136 | 136 | 3311 | 311 | 4557 | 557 |
| Bonf.corr.MSEmedian | 998 | -2 | 1997 | -3 | 2982 | -18 | 3997 | -3 |
| Bonf.fitfZ.conv | 1007 | 7 | 2012 | 12 | 3041 | 41 | 4127 | 127 |
| truncfdr | 978 | -22 | 1729 | -271 | 2331 | -669 | 2765 | -1235 |
| truncfdr.corr | 1051 | 51 | 2135 | 135 | 3363 | 363 | 4613 | 613 |
| truncfdr.corr1 | 1042 | 42 | 2083 | 83 | 3245 | 245 | 4431 | 431 |
| truncfdr.corr2 | 1058 | 58 | 2175 | 175 | 3446 | 446 | 4719 | 719 |
| truncfdr.corr.median | 1032 | 32 | 2029 | 29 | 3097 | 97 | 4144 | 144 |
| truncfdr.corr.MSEmedian | 1017 | 17 | 1939 | -61 | 2864 | -136 | 3731 | -269 |
| truncfdr.fitfZ.conv | 1037 | 37 | 2027 | 27 | 3076 | 76 | 4119 | 119 |
