# Supplementary material for: Estimating the Total Number of Susceptibility Variants Underlying Complex Diseases from Genome-Wide Association Studies
Source: PLoS One. 2010 Nov 17;5(11):e13898. doi: 10.1371/journal.pone.0013898 (PMC2984437; doi:10.1371/journal.pone.0013898)
Supplement: Table S3 — SD of different estimators from simulations. (0.07 MB DOC) [file pone.0013898.s004.doc]

Table S3 SD of different estimators from simulations

|  | λ=1000 | λ=2000 | λ=3000 | λ=4000 |
| --- | --- | --- | --- | --- |
| *N*=3500 |  |  |  |  |
| Bonf | 52 | 98 | 138 | 191 |
| Bonf.corr | 125 | 482 | 1099 | 2288 |
| Bonf.corr1 | 117 | 461 | 1122 | 2615 |
| Bonf.corr2 | 131 | 480 | 996 | 1803 |
| Bonf.corr.med | 107 | 387 | 942 | 2206 |
| Bonf.corr.MSEmedian | 93 | 318 | 786 | 1868 |
| Bonf.fitfZ.conv | 91 | 349 | 992 | 3585 |
| truncfdr | 49 | 92 | 114 | 144 |
| truncfdr.corr | 81 | 278 | 511 | 867 |
| truncfdr.corr1 | 78 | 269 | 515 | 896 |
| truncfdr.corr2 | 83 | 278 | 480 | 777 |
| truncfdr.corr.median | 71 | 222 | 409 | 704 |
| truncfdr.corr.MSEmedian | 63 | 179 | 324 | 551 |
| truncfdr.fitfZ.conv | 72 | 239 | 500 | 1119 |
|  |  |  |  |  |
|  |  |  |  |  |
| *N*=5000 |  |  |  |  |
| Bonf | 58 | 102 | 140 | 184 |
| Bonf.corr | 101 | 353 | 779 | 1319 |
| Bonf.corr1 | 96 | 329 | 759 | 1307 |
| Bonf.corr2 | 106 | 367 | 757 | 1241 |
| Bonf.corr.med | 93 | 292 | 639 | 1130 |
| Bonf.corr.MSEmedian | 84 | 242 | 530 | 909 |
| Bonf.fitfZ.conv | 82 | 245 | 564 | 1134 |
| truncfdr | 54 | 98 | 140 | 148 |
| truncfdr.corr | 73 | 214 | 459 | 624 |
| truncfdr.corr1 | 71 | 206 | 446 | 624 |
| truncfdr.corr2 | 75 | 218 | 454 | 594 |
| truncfdr.corr.median | 67 | 182 | 372 | 485 |
| truncfdr.corr.MSEmedian | 63 | 156 | 302 | 383 |
| truncfdr.fitfZ.conv | 68 | 179 | 392 | 589 |
|  |  |  |  |  |
|  |  |  |  |  |
| *N*=7000 |  |  |  |  |
| Bonf | 52 | 102 | 139 | 183 |
| Bonf.corr | 73 | 251 | 526 | 947 |
| Bonf.corr1 | 70 | 235 | 496 | 924 |
| Bonf.corr2 | 76 | 262 | 537 | 918 |
| Bonf.corr.med | 69 | 219 | 452 | 846 |
| Bonf.corr.MSEmedian | 65 | 191 | 381 | 717 |
| Bonf.fitfZ.conv | 64 | 180 | 355 | 667 |
| truncfdr | 55 | 106 | 142 | 175 |
| truncfdr.corr | 67 | 177 | 326 | 541 |
| truncfdr.corr1 | 66 | 171 | 313 | 526 |
| truncfdr.corr2 | 68 | 181 | 331 | 537 |
| truncfdr.corr.median | 65 | 159 | 289 | 465 |
| truncfdr.corr.MSEmedian | 62 | 144 | 249 | 387 |
| truncfdr.fitfZ.conv | 63 | 156 | 272 | 455 |
