# Supplementary material for: Estimating the Total Number of Susceptibility Variants Underlying Complex Diseases from Genome-Wide Association Studies
Source: PLoS One. 2010 Nov 17;5(11):e13898. doi: 10.1371/journal.pone.0013898 (PMC2984437; doi:10.1371/journal.pone.0013898)
Supplement: Table S4 — Estimates and confidence intervals of lambda for a few complex traits, with inclusion threshold based on Bonferroni correction. (0.04 MB DOC) [file pone.0013898.s005.doc]

Table S4 Estimates and confidence intervals of lambda for a few complex traits, with inclusion threshold based on Bonferroni correction

|  | HDL | LDL | TG | DM(all SNPs) | DM(pruned) | Crohn(all SNPs) | Crohn(pruned) |
| --- | --- | --- | --- | --- | --- | --- | --- |
| **Estimates of lambda** |  |  |  |  |  |  |  |
| Bonf | 371 | 525 | 456 | 398 | 582 | 784 | 1105 |
| Bonf.corr | 432 | 657 | 560 | 557 | 1140 | 1119 | 2113 |
| Bonf.corr1 | 424 | 645 | 547 | 530 | 1071 | 1071 | 2067 |
| Bonf.corr2 | 439 | 664 | 570 | 578 | 1182 | 1156 | 2103 |
| Bonf.corr.med | 411 | 620 | 524 | 503 | 976 | 1005 | 1814 |
| Bonf.corr.MSEmedian | NA | NA | NA | 480 | 855 | 939 | 1652 |
| Bonf.fitfZ.conv | 395 | 591 | 499 | 518 | 922 | 946 | 1520 |
| **Confidence intervals** |  |  |  |  |  |  |  |
| upCI (Bonf) | 444 | 665 | 547 | 704 | 1885 | 1108 | 2201 |
| loCI (Bonf) | 346 | 516 | 450 | 332 | -40 | 783 | 840 |
| upCI.MLRT (Bonf) | 447 | 669 | 549 | 719 | 2538 | 1120 | 2343 |
| loCI.MLRT (Bonf) | 348 | 520 | 452 | 364 | 295 | 794 | 953 |
